# Pathological Roles of Mitochondrial Oxidative Stress and Mitochondrial Dynamics in Cardiac Microvascular Ischemia/Reperfusion Injury

**DOI:** 10.3390/biom10010085

**Published:** 2020-01-05

**Authors:** Hao Zhou, Sam Toan

**Affiliations:** 1 Department of Cardiology, Chinese PLA General Hospital, Medical School of Chinese PLA, Beijing 100853, China; 2 Department of Chemical Engineering, University of Minnesota-Duluth, Duluth, Minnesota, MN 55812, USA

**Keywords:** microvascular I/R injury, endothelial cells, mROS, mitochondrial dynamics

## Abstract

Mitochondria are key regulators of cell fate through controlling ATP generation and releasing pro-apoptotic factors. Cardiac ischemia/reperfusion (I/R) injury to the coronary microcirculation has manifestations ranging in severity from reversible edema to interstitial hemorrhage. A number of mechanisms have been proposed to explain the cardiac microvascular I/R injury including edema, impaired vasomotion, coronary microembolization, and capillary destruction. In contrast to their role in cell types with higher energy demands, mitochondria in endothelial cells primarily function in signaling cellular responses to environmental cues. It is clear that abnormal mitochondrial signatures, including mitochondrial oxidative stress, mitochondrial fission, mitochondrial fusion, and mitophagy, play a substantial role in endothelial cell function. While the pathogenic role of each of these mitochondrial alterations in the endothelial cells I/R injury remains complex, profiling of mitochondrial oxidative stress and mitochondrial dynamics in endothelial cell dysfunction may offer promising potential targets in the search for novel diagnostics and therapeutics in cardiac microvascular I/R injury. The objective of this review is to discuss the role of mitochondrial oxidative stress on cardiac microvascular endothelial cells dysfunction. Mitochondrial dynamics, including mitochondrial fission and fusion, are critically discussed to understand their roles in endothelial cell survival. Finally, mitophagy, as a degradative mechanism for damaged mitochondria, is summarized to figure out its contribution to the progression of microvascular I/R injury.

## 1. Introduction

Acute myocardial infarction (AMI) is caused by blockage of one or more of the coronary arteries that supply the heart [1,2]. The sudden cessation of fresh blood flow will extensively result in tissue hypoxia or anoxia and, eventually, cell death through apoptosis or necrosis [3,4]. It has been widely accepted that re-introduction of blood flow through reperfusion strategies is necessary and fruitful to salvage damaged myocardium. Paradoxically, reperfusion also causes cardiomyocyte or endothelial cell death through inducing oxidative stress, calcium overload, and tissue inflammation response [5,6]. Of note, the clinical reality of ischemia-reperfusion (I/R) injury becomes apparent with the advent of thrombolytic and interventional reperfusion [7,8].

The cardiac circulation is not only the culprit of acute myocardial infraction through coronary occlusion due to the formation of thrombus after plaque erosion or rupture but also a victim of reperfusion treatment after myocardial ischemia [9,10]. Structurally, endothelial cells constitute a layer between blood and extravascular cells and are responsible for maintaining the structure and regulating the function of blood vessels [11,12]. In addition to regulating the vascular tone, a series of dilators (such as bradykinin and nitric oxide) and constrictors (like endothelin) are generated, released, or regulated by endothelial cells, in order to prevent platelet aggregation and blood clot formation [13,14]. In the heart, endothelial cells sense the alterations of micro-environment and then release of a number of cell signaling transmitters to maintain hemostasis in the vessel and heart tissue [15,16]. Under normal conditions, no adhesion molecules or thrombogenic factors are expressed on the surface of endothelial cells; however, when blood vessels are damaged, vascular endothelial growth factor (VEGF) and adhesion molecule are expressed on the surface of endothelial cells and contribute to the pro-inflammation cells accumulation [17,18]. This effect may aggravate microvascular stenosis, especially at the stage of reperfusion [19,20]. From a clinical point of view, no-reflow phenomenon with severe capillary damage occurs in 25–50% of patients that received reperfusion strategies including percutaneous transluminal coronary intervention or coronary artery bypass grafting surgery [21,22]. More importantly, no-reflow phenomenon negatively affects the clinical outcome in patients with AMI and is highly correlated with arrhythmias, left ventricular remodeling, and death in the short, medium, and long term [23,24]. In addition, no-reflow phenomenon, mainly caused by cardiac microvascular I/R injury, has been used as an independent predictor of mortality at 5 years [25,26]. Unfortunately, although many studies have been performed to understand the molecular mechanisms underlying cardiomyocyte I/R injury, cardiovascular microvascular I/R injury is a so far neglected target of cardioprotection [27,28].

**Figure 1:**
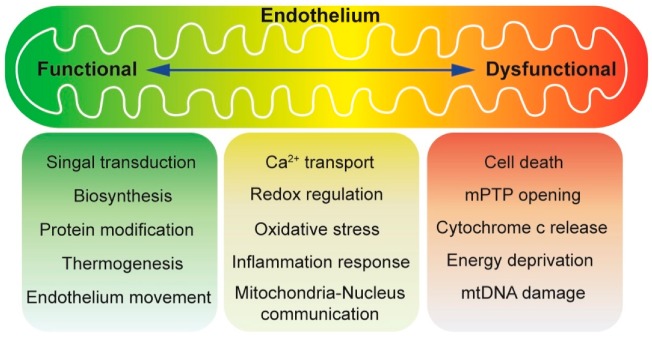
An overview of mitochondrial function in endothelium homeostasis. Mitochondria are known as the powerhouse of the cell. Under normal conditions, mitochondria-dependent energy production is relatively low in vascular endothelium, which primarily uses glycolysis to produce ATP. It is now accepted that mitochondria in endothelial cells mainly play a prominent role in signaling cellular responses to environmental cues. Intermediate metabolism in the mitochondria produces metabolites for biosynthesis, protein modification, and thermogenesis. In addition, endothelial mobilization is under the control of mitochondria. Oxidative phosphorylation is coupled with generation of reactive oxygen species (ROS), which can either serve as molecular signals or cause cell damage and cell death. Mitochondrial metabolism is stimulated by calcium, but under pathological conditions, calcium overload can trigger the opening of the mitochondrial permeability transition pore (mPTP). The release of mitochondrial content, such as cytochrome c, induces apoptosis, or the loss of membrane potential (a consequence of prolonged mPTP opening), causes ATP deprivation and necrosis.

Mitochondria have historically been viewed as the battery of the cell through consuming oxygen and producing ATP with the help of citric acid cycle [29,30]. A host of cellular stress responses are under the control of mitochondria in addition to their necessary role in bioenergetics [29,31]. Unlike cardiomyocytes or skeletal muscle, mitochondria-dependent energy production is relatively low in vascular endothelium, which primarily uses glycolysis to produce ATP [32,33]. It is now accepted that mitochondria in endothelial cells mainly play a prominent role in signaling cellular responses to environmental cues [34,35]. More importantly, mitochondrial content in endothelial cells is relatively low (2–6% of cytoplasm volume) compared with other cell types such as cardiomyocytes (~32%) [36]. The low mitochondrial content in endothelial cells further validates a non-canonical function played by mitochondria in regulating signaling responses rather than in glucose metabolism [37]. In response to stress, reactive oxygen species (ROS) are produced by mitochondria and employed as a second messenger to transduce extracellular signal [38,39]. Mitochondrial fusion [40] and fission [41,42], together with mitophagy (removal of defected mitochondria), are also involved in the regulation of cell homeostasis through affecting the mitochondrial quality control [43,44]. Here, we summarize and discuss the main regulatory aspects of mitochondrial oxidative stress and mitochondrial dynamics in cardiac microvascular I/R injury (Figure 1).

## 2. Mitochondrial Oxidative Stress

Oxygen is carried in from circulating blood transfers to perivascular tissues through endothelial cells. Of note, endothelial cells consume a slight amount of oxygen to produce ATP in order to transfer most of the oxygen to myocardium [45,46]. In vitro data suggest superoxide generated by mitochondria accounts for 0.2–2% of cellular oxygen consumption [47,48]. It has been found that endothelial cell dysfunction affects the diffusion rate of oxygen across the arteriolar vessel wall [49,50]; however, these observations require further discussion. In endothelial cells, due in larger part to the unique nature of endothelium’s limited metabolic demands, mtROS primarily play a prominent role as cell-signaling molecules [51,52]. Vascular endothelial cells overwhelmingly rely on glycolysis rather than the TCA cycle for cellular ATP needs [53,54]. This allows endothelial cells the freedom to leverage the products of the electron transport chain (ETC) for cell signaling purposes. Jensen first observed the formation of ROS in the respiration chain [55,56]. This point has been further validated by later studies that confirm mtROS production mainly takes place at the electron transport chain localized on the inner mitochondrial membrane during the process of oxidative phosphorylation [57,58]. Mechanistically, the primary sites of superoxide anion production and release are Complex I (NADH-ubiquinone oxidoreductase), Complex II (succinate dehydrogenase, SDH), and Complex III (ubiquinol-cyctochrome c oxidoreductase) [59]. Complexes I and II accept electrons from NADH + H^+^ and FADH_2_, respectively, which are transferred to Complex III and finally to Complex IV (cytochrome c oxidase), where the final electron acceptor is oxygen and the final product is water [60]. In the ETC, Complex I and Complex III are primarily responsible for producing superoxide anions (O_2_^−^). Complex I produces O_2_^−^ in the process of oxidizing NADH into NAD and pumping a proton from the mitochondrial matrix into the intermembrane space [53]. Complex III produces O_2_^−^ as it oxidizes CoQ to reduce cyt-c and pumps a proton into the intermembrane space [61,62]. Molecules of O_2_^−^ are potent oxidants and, therefore, need to be reduced and “detoxified”.

NADPH oxidase (NOX) is another source of mtROS production in endothelial cells. NOX are membrane-bound enzyme complexes and NOX subunits gp91^phox^, p22^phox^, p67^phox^, and p47^phox^ were first identified in cultured HUVEC by Jones and colleagues [63]. These NOX isoforms expressed in the vasculature differ in their subcellular localization, yet all function through electron transfer from cytosolic NOX to mitochondrial oxygen, thereby producing superoxide or hydrogen peroxide [64,65]. Although ample evidence in various endothelial cell lines has indicated mitochondrial ETC as the main source of I/R-related ROS, there is a possibility of cross-talk between NOX isoforms and mitochondria [66]. Of note, NOX4 is localized along the inner mitochondrial membrane, and the potential interaction between these two sources of ROS may be significant determinants of the total mtROS in the endothelium [67,68].

In addition to mitochondria and NOX, ROS in endothelial cells are also generated by xanthine oxidase and neutrophils, especially at the stage of I/R injury [69]. Xanthine oxidase, the conversion product of oxidative damage to xanthine dehydrogenase, produces superoxide and hydrogen peroxide during purine metabolism [70,71]. This process is more prominent in endothelial cells [72]. First, xanthine oxidase is abundant in endothelial cells [64]. Second, xanthine oxidase-mediated ROS formation is a byproduct of ATP metabolism independent of mitochondria-related oxidative phosphorylation [73,74]. Third, xanthine oxidase is primarily activated by reperfusion after ischemia—hypoxia breaks down ATP and then forms a lot of AMP/hypoxanthine, which is utilized to generate ROS once oxygen is re-introduced [75]. These ROS recruit neutrophils to the blood-endothelial cell interface, thereby initiating migration into the surrounding tissues. Neutrophils then produce a greater amount ROS, further precipitating the effects of I/R injury [56,76]. In the normal physiological state, multiple antioxidant mechanisms exist to counteract the effect of mtROS: Superoxide dismutase (SOD), glutathione, and catalase [77,78]. Oxidative stress in endothelial cells is the result of increased ROS production and depressed antioxidant system [72]. The predominant ROS in the endothelial cells are superoxide and hydroxyl radicals. Their cellular toxicity comes from lipid peroxidation and its associated membrane damage [79,80]. DNA damage, oxidative post-transcriptional modification of cysteine residues in protein, and signal transduction are also governed by mtROS [81,82]. Due to the instability of free radical species, free radical scavengers have been utilized to directly prove the detrimental effects of mtROS in cardiac microvascular I/R injury.

In the mitochondrial matrix, superoxide dismutase (SOD) 2 reduces O_2_^−^ into H_2_O_2_, a less toxic ROS [83]. Then, glutathione peroxidase (GPX) catalyzes the reduction of H_2_O_2_ into H_2_O through the oxidation of reduced glutathione (GSH) into its oxidized form (GSSG) [84,85]. Catalase in the mitochondrial matrix can also convert H_2_O_2_ into water and molecular oxygen [86,87]. Glutathione biosynthesis is catalyzed by glutathione reductase using the oxidation of reduced nicotinamide adenine dinucleotide phosphate (NADPH) into NADP and is crucial in antioxidant activities in the mitochondria [88,89]. In the intermembrane space, copper and zinc-containing SOD1 reduces O_2_^−^ into H_2_O_2_, and GPX reduces H_2_O_2_ into H_2_O [90,91].

Another important source of ROS in the re-perfused heart are the two isoforms of monoamine oxidases (MAO), MAO-A and MAO-B, which are located on the outer mitochondrial membrane [92]. It has been demonstrated that MAO-A activity is enhanced by I/R injury and is responsible for the precipitation of hydrogen peroxide and the progression towards left ventricle hypertrophy and cardiac remodeling [93]. Increased influx of mitochondrial iron stimulates the formation of more potent and deleterious hydroxyl radical groups from hydrogen peroxide [94,95]. Although the continuous release of ROS from mitochondria during normal conditions appears to play a necessary role in the maintenance of basal cellular function, transiently elevated ROS levels can promote selective protein synthesis, preconditioning, and changes in vascular tone [96,97]. However, even modest, acutely elevated mitochondrial ROS production can lead to cellular dysfunction.

Under I/R injury, endothelial cell proliferation is delayed due to excessive mtROS production [98]. Endothelial cell migration and tube formation are also impaired, an effect that is accompanied with a decrease in the expression of VEGF, VE-cadherin, and MMP2 [98,99], suggesting that oxidative stress affects the angiogenesis and regenerative capacity of micro-vessels in reperfused myocardium. Mechanistically, mtROS overproduction seems to be associated with a decrease in the levels of total antioxidant capacity and SOD [100]. Of note, the increase in mtROS in endothelial cells under I/R injury may also result from excessive inflammation response because myeloperoxidase (MPO) expression, a neutrophil infiltration marker, is upregulated in reperfused heart [101,102]. Increased thioredoxin-interacting protein (TXNIP) and a noticeable decline in the levels of thioredoxin 1 (Trx-1), thioredoxin reductase (TrxR), glutathione (GSH), catalase (CAT), and glutathione peroxidase (GPx) may also contribute to endothelial oxidative stress [103]. In order to attenuate oxidative stress in endothelial cells under I/R injury, several drugs or approaches have been developed. Ginkgolide A (GA) [104] and liraglutide [105] have anti-oxidative properties and could reduce endothelial oxidative stress, attenuating microvascular damage induced by reperfusion. Peroxisome proliferator-activated receptor γ (PPAR-γ) agonist inhibits vascular complications in I/R injury through modulation of oxidative stress and endoplasmic reticulum stress [106]. A histone deacetylase 7-derived peptide maintains endothelium integrity and promotes angiogenesis in hindlimb ischemia partly through regulation of oxidative stress [107]. Propylene glycol alginate sodium sulfate (PSS) as heparinoid drug has many biological activities. A novel PSS-loaded nanoparticle has been found to normalize oxidative stress and thus reverse coronary microcirculation dysfunction [108,109]. 

Of note, oxidative stress is associated with altered expression of mitochondrial and nuclear proteins. For instance, it has been demonstrated that oxidative stress increases the activity of COUP-TFII transcription factor, which induces the expression of nuclear-encoded mitochondrial enzymes, favoring mitochondrial fragmentation [110]. Similarly, ROS downregulate the activity of ETC complexes and decrease oxygen consumption in patients with metabolic syndrome, which results in left ventricular hypertrophy and heart failure [111]. Lastly, ROS provoke structural changes in mitochondrial proteins, such as an imbalance between mitochondrial tyrosine kinase Src and phosphatase SPH2, which decreases tyrosine phosphorylation at the active region of many mitochondrial enzymes [112]. In cardiac microvascular I/R injury, inhibition of ROS generation has been found to reverse the transcription and expression of survivin [113], an anti-apoptotic protein. 

Last but not least, the deterioration of mtROS is responsible for triggering cell death/loss during cardiac I/R. It is reported that biphasic mtROS dynamics may occur, which include gradual mtROS increase followed by mtROS flash. Of note, baseline mtROS increase and accumulation could be an activation signal for mtROS flash, which has been defined as a well-known and important phenomenon of ROS-induced ROS-release, first described by Zorov et al. in cardiomyocytes [114]. However, this point has not been validated in cardiac microvascular I/R injury. In addition, the molecular mechanism underlying baseline mtROS elevation and accumulation remains unknown.

## 3. Mitochondrial Fission 

**Figure 2:**
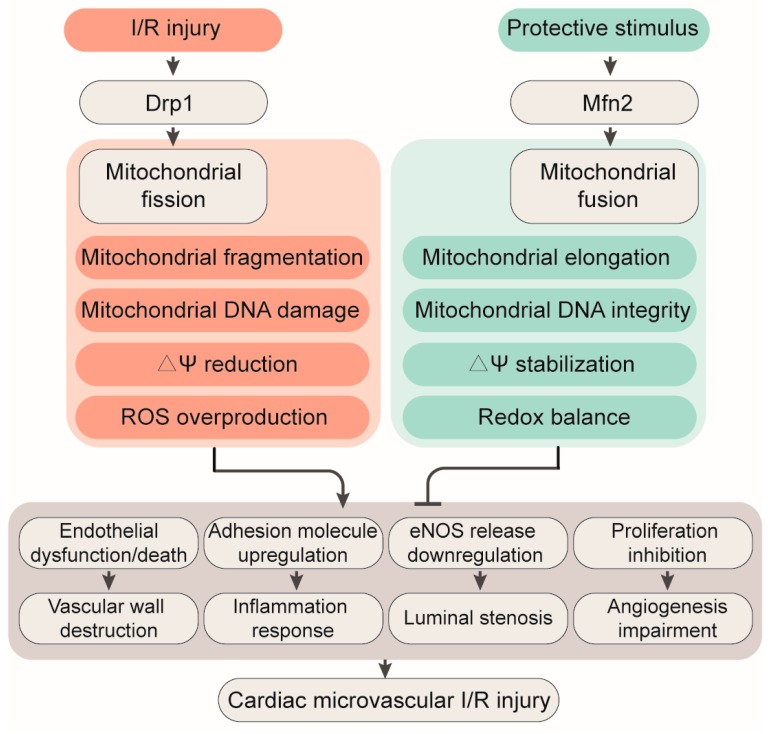
Role of mitochondrial fission and fusion in cardiac microvascular ischemia/reperfusion (I/R) injury. Increased mitochondrial fission is followed by mitochondrial damage, proliferation inhibition, apoptosis, and vascular inflammation. In contrast, mitochondrial fusion increases the resistance of cardiac microcirculation against I/R injury.

Mitochondria are dynamic organelles, not static entities. Mitochondria can undergo replication, fission, and fusion; move from one location to another within cells; and form networks with other mitochondria or cellular structures to increase efficiency of ATP production as well as providing for intracellular signaling in response to physiological and pathological stimuli [115,116]. Increased fusion or reduced fission promotes the formation of elongated mitochondrial networks, whereas increased fission or reduced fusion causes mitochondrial fragmentation [117]. Cells that primarily use mitochondria metabolism to generate ATP, such as cardiomyocytes, have more fusion and more elongated mitochondrial networks [118], whereas the mitochondria in cells that are more glycolytic and less reliant on mitochondria-mediated ATP synthesis, such as microvascular endothelial cells, appear more punctate [119]. Mechanistically, mitochondria undergo membrane remodeling through dynamin-related protein 1 (Drp1) [120]. Drp1 is widely and diffusely disturbed throughout the cytosol under normal condition and translocates to the outer mitochondria membrane when activated via posttranslational modifications (predominantly phosphorylation/dephosphorylation) [121]. Once positioned on the outer mitochondrial membrane, Drp1 interacts with four mitochondrial-bound proteins that serve as Drp1 receptors (mitochondrial dynamic proteins of 49 and 51 kDa (Mid49 and Mid51), mitochondrial fission protein 1 (Fis1), and mitochondrial fission factor (Mff), where it constricts and cleaves the mitochondria [122] (Figure 2). 

Under physiological condition, fission is required for cell division and movement of mitochondria within the cell and is involved in the elimination of senescent mitochondria. Fission is also an adaptive response to cellular stress that facilitates the isolation and removal of damaged mitochondrial components by mitophagy [123,124]. Finally, mitochondrial fission occurs concomitantly with outer membrane permeabilization and release of cytochrome c during apoptosis, although it remains controversial whether fission is a necessary step in mitochondria-dependent apoptosis [125,126]. Under I/R injury, Drp1 is primarily phosphorylated at Ser616 and dephosphorylated at Ser637 [127], resulting in Drp1 accumulation around mitochondria outer membrane to form a potential contractile ring. Although the role of mitochondrial fission in cardiomyocyte I/R injury has been widely explored [128,129,130], the regulatory mechanisms and functional contributions of fission in endothelial I/R injury remain unclear. Based on recent studies [127,131,132,133,134,135,136], mitochondrial fission affects endothelial cell fate possibly through three mechanisms. First, excessive mitochondrial fission causes mitochondrial DNA damage, as evidenced by mitochondrial DNA double strand breakage. This effect may blunt mitochondrial DNA copying and transcription. Due to an indispensable role played by mitochondrial DNA in regulating the expression and activity of mitochondrial respiratory complex, mitochondrial DNA damage is associated with mitochondrial respiration dysfunction, leading to proton leak and ROS formation. Second, the accumulation of mtROS induces mitochondrial lipid oxidation, especially cardiolipin. Peroxided cardiolipin loses its affinity to cyt-c, resulting in cyt-c detachment from mitochondrial inner membrane, an early marker of cell apoptosis. Third, abnormal mitochondrial fission induces mitochondrial fragmentation, and this effect mediates voltage-dependent anion-selective channel 1 (VDAC1) multimerization, a prerequisite for mPTP opening, which is a feature of mitochondria-initiated cell necrosis or necroptosis. Likely through the above three mechanisms, mitochondrial fission exacerbates endothelial cells’ oxidative stress, induces mitochondrial DNA damage, and activates mitochondria-related cell death. Later studies have further found that mitochondrial fission in endothelial cells is activated or inhibited by nuclear receptor subfamily 4 group A member 1 (NR4A1) [135] or Bax inhibitor-1 (BI-1) [131,136], respectively. NR4A1 induces Mff phosphorylation and enhances Drp1 recruitment onto mitochondria [135], leading to mitochondrial fragmentation. In contrast, BI-1 interrupts the ROS-mediated mitochondrial damage and partly blocks mitochondrial fission-induced endothelial cell death [131,136]. However, the relationship between mitochondrial fission and oxidative stress and the threshold of physiological mitosis shift to fatal mitochondrial fission require further investigation.

## 4. Mitochondrial Fusion 

Mitochondria may be separate or found in a network, where permanent dynamic fission and fusion can occur [137]. I/R injury or endothelial oxidative stress may alter mitochondrial shape, movement, and cellular interactions [138]. Mitochondria form a dynamic interconnected intracellular network, changing cellular location through cytoskeletal motors and altering size and shape in response to the metabolic needs of the cells. Fusion is mediated by three different GTPases: Optic atrophy 1 (Opa1), mitofusin 1 (Mfn1), and mitofusin 2 (Mfn2) [139,140] (Figure 2). Both Mfn1 and Mfn2 mediate fusion of the outer mitochondrial membranes [141], while Opa1 mediates the fusion of the inner mitochondrial membrane [142]. Mechanistically, mitofusins interact and form a hemifusion stalk to initiate the joining of two mitochondrial membranes [143]. The stalk then grows and creates a lipidic hole as well as a hemifusion diaphragm to reestablish membrane continuity [144]. Finally, a fusion pore is made for inner membrane fusion via the lipid binding domain in Opa1 that is specific for cardiolipin. Fusion of the inner and outer mitochondrial membrane is regulated through proteolytic cleavage and ubiquitination, respectively [145]. Opa1 consists of eight different isoforms generated by alternative splicing of three of the 30 Opa1 exons. Membrane-bound long Opa1 (L-Opa1) could be processed via two proteolytic cleavage sites (S1 and S2), generating short forms (S-Opa1) [146,147]. Proteolytic processing is carried out predominantly through two intermembrane space AAA proteases (ATPases associated with diverse cellular activities): (i) overlapping with m-AAA (OMA1) cleaving at the S1 site and (ii) yeast mitochondrial DNA escape 1-like (YME1L) cleaving at the S2 site [148,149,150]. Under normal physiological conditions, S1 and S2 are constitutively cleaved to produce a 50:50 ratio of L-Opa1 and S-Opa1 [151,152]. However, after exposure to stressful conditions, such as mitochondrial membrane depolarization, low levels of ATP, or oxidative stress, the balance is tipped and most L-Opa1 are cleaved by Oma1 resulting in mitochondrial fragmentation [153,154]. With respect to ubiquitin, mitofusins are primarily regulated by ubiquitin-mediated degradation, specifically through the PTEN-induced kinase (PINK1) and Parkin-mediated ubiquitination pathway [155]. This pathway is closely associated with mitophagy and is discussed later in further detail. 

Under physiological conditions, fusion and fission are balanced, and mitochondrial networks are present. Fusion facilitates distribution of metabolites, proteins, and mtDNA and helps maintain electrical and biochemical connectivity [156,157,158]. However, there is little evidence available to describe the precise role played by mitochondrial fusion in microvascular I/R injury. In hyperhomocysteinemia-treated endothelial cells, mitochondrial fission is increased whereas fusion is inhibited, leading to ROS overproduction and endothelial cell death [159]. After ablation of toll-like receptor 4, reverse mitochondrial fusion thus reduces endothelial dysfunction during hyperhomocysteinemia [160]. Fluid mechanical forces have been found to regulate mitochondrial fusion activity in a manner dependent on intracellular calcium concentration [161]. Considering that fluid shear stress may take place at the initial stage of reperfusion, it is of interest to explore the influence of fluid shear stress on mitochondrial fusion. In a mouse model of type-1 diabetes, the levels of Opa1 are downregulated, indicative of mitochondrial fusion inactivation. Interestingly, administration of ROS scavenger TEMPOL leads to a significant decrease in mitochondrial fragmentation without altering the levels of Opa1 [162]. These results indicate that mitochondrial fusion may play a protective role in regulating endothelial cell function. Further works are required to figure out the primary adaptors underlying mitochondrial fusion regulation in endothelial cells under I/R injury.

## 5. Mitophagy 

**Figure 3:**
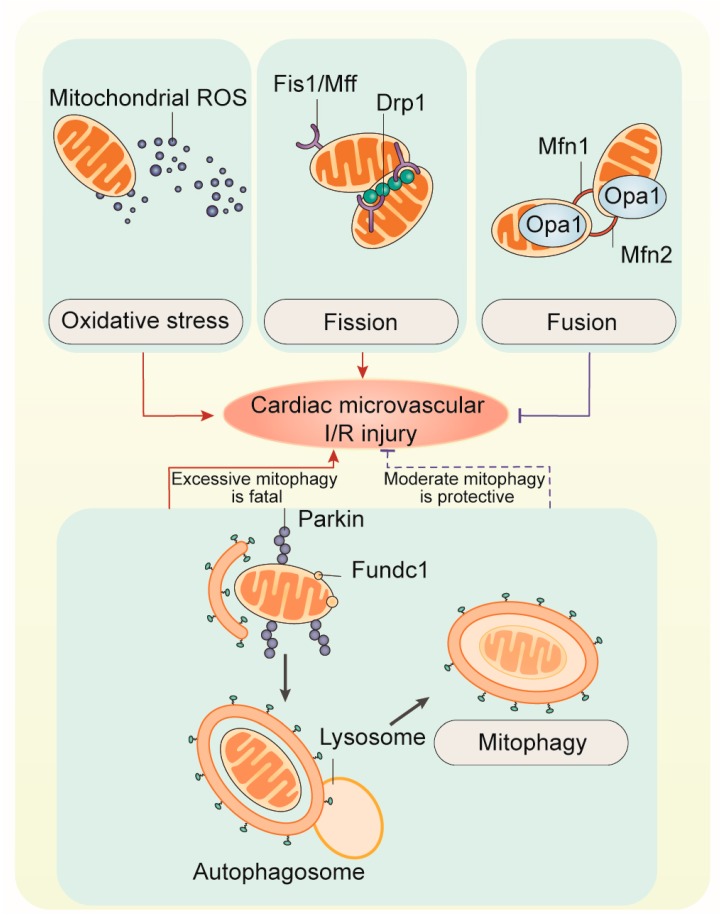
Overview of mitochondrial oxidative stress and mitochondrial dynamics in cardiac microvascular I/R injury. The mitochondrial network is constantly reshaped by the antagonistic activity of proteins that mediate fission, such as mitochondrial fission factor (Mff), mitochondrial fission 1 protein (Fis1), and dynamin 1-like protein (Drp1), and proteins that promote fusion, such as mitofusin 1 (Mfn), Mfn2, and optic atrophy protein 1 (Opa1). One of the essential roles of fission is to segregate dysfunctional mitochondria, thereby enabling their uptake by the autophagic machinery and consequent degradation in lysosomes. Parkin, parkin RBR E3 ubiquitin protein ligase; PINK1, PTEN-induced putative kinase protein 1; Fundc1, Fun14 domain-containing protein 1.

Mitochondrial dysfunction causes cell/organ injury through several mechanisms, including diminished cellular energy status (low cellular ATP level, energy stress) and enhanced production of reactive oxygen species (ROS). Furthermore, mitochondrial damage is associated with the release of several apoptosis-activated factors, leading to programmed cell death [163]. Disturbances in ionic balance, particularly an increase in mitochondrial and cytoplasmic Ca^2+^, stimulates mitochondrial permeability transition (PT) accompanied by the opening of non-selective channels known as the PT pores (PTP) that allow free movement of ions and other solutes with a molecular mass <1.5 kDa across the inner mitochondria membrane (IMM) [4]. As a result, mPTP opening enhances colloid-osmotic pressure in the matrix, leading to mitochondrial swelling associated with the activation of proteases and lipases that eventually lead to cell death [164] (Figure 3).

Mitophagy is the selective degradation of damaged mitochondria by autophagy. In this process, mitochondria are sequestered in autophagosomes and delivered to lysosomes for hydrolytic degradation [165]. Physiologically, mitophagy plays essential roles in development, including the complete removal of damaged mitochondria to keep mitochondrial network homeostasis [166]. Abnormal mitophagy exacerbates mitochondrial damage and cell death through inducing ATP depletion and mitophagy-dependent necrosis or mitophagic cell death [167,168]. Like autophagy, mitophagy shares the core molecular machinery with autophagy, which is initiated by the nucleation of an isolation membrane, and then the isolation membrane elongates and closes to form an autophagosome [169]. The origin of autophagosome membranes still remains controversial whereas autophagosome formation is regulated by two ubiquitin-like conjugation systems [170,171,172], Atg12-Atg5 and Atg8-PE. However, in contrast to mitophagy, autophagy is considered as a nonselective bulk degradative process where the autophagosomes randomly engulf contents in the cytosol [173,174]. Mitophagy induction and regulation are regulated by receptor-dependent or -independent pathways. 

The most recognized mitophagy pathway in mammalian cells is mediated by PINK1 and Parkin, a receptor-independent pathway [175]. PINK1, a serine/threonine kinase, is constitutively imported to the inner membrane through its mitochondrial target sequence. Under normal condition, PINK1 is primarily cleaved by the inner membrane presenilin-associated rhomboid-like protease PARL and ultimately proteolytically degraded [176]. Upon mitochondria damage, such as mitochondrial membrane potential depolarization, PINK1 degradation is suppressed, and thus the full length of PINK1 is accumulated on the mitochondrial outer membrane [177]. Subsequently, PINK1 recruits Parkin from the cytosol to mitochondria [178]. Upon localization onto mitochondria, Parkin ubiquitinates mitochondrial membrane proteins such as mitofusins [179]. p62 is also recruited by Parkin to ubiquitinated mitochondria to promote the delivery of ubiquitinated mitochondria to autophagosome via the binding to LC3. Of note, Parkin can also interact directly with autophagy-regulating proteins such as Ambra1 to facilitate mitophagy [180].

In addition to PINK1/Parkin-mediated mitophagy, receptor-dependent pathways for mitophagy induction include Bnip1, Nix, and Fundc1 [181]. BCL2 and adenovirus E1B 19-kDa-interacting protein 3 (BNIP3) and BNIP3-like (BNIP3L or Nix) are Bcl2 family proteins with an atypical BH3 domain [182]. Fun14 domain-containing protein 1 (Fundc1) is a mitochondrial outer membrane protein containing a conserved LC3 interaction region (LIR) with a W/Y/FxxL/I/V motif [183]. BNIP3 and Nix were initially identified as pro-death proteins and were recently identified as mitophagy activators in specific conditions. Nix seems indispensable for the complete mitochondrial elimination during reticulocyte maturation [184]. In Nix-deficient mice, mitochondrial clearance in reticulocytes was dramatically inhibited or retarded [184]. Several studies also revealed that Nix deficiency inhibits mitochondrial depolarization, and treatment with uncoupling chemicals (e.g., CCCP) or a BH3 mimetic (e.g., ABT-737) is able to induce mitochondrial depolarization and then restore the sequestration of mitochondria into autophagosomes in Nix-deficient erythroid cells, suggesting that one mechanism for Nix induced-mitophagy is inducing mitochondrial depolarization [185]. Unlike Nix, BNIP3 and Fundc1 have also been implicated in hypoxia-induced mitophagy [130,135]. Under hypoxia conditions, BNIP3 expression is increased along with translocation onto the surface of mitochondria [130], whereas Fundc1 is primarily dephosphorylated at different sites including Ser13, Ser18, and Tyr17 [41,186,187,188]. Interestingly, BNIP3-mediated mitophagy is always followed by cell death [130], whereas Fundc1-related mitophagy mitigates cell death through preventing ROS overproduction and mitochondrial depolarization [1,35].

In endothelial cells under I/R injury, mitophagy seems to be modulated by Sirt3 in a manner dependent on PINK1-Parkin pathway [189]. Loss of Sirt3 facilitates angiotensin II-induced aberrant PINK1/Parkin acetylation and impairs mitophagy, and then excessive mtROS generation limits angiogenic capacity in primary mouse cardiac microvascular endothelial cells [189]. Of note, endothelial mitophagy during hypoxia is primarily regulated by endothelial uncoupling protein 2 (Ucp2). Unlike Sirt3, Ucp2 endothelial knockout mice lead to excessive PINK1/Parkin-related mitophagy, inadequate mitochondrial biosynthesis, and increased apoptosis in endothelium [190], suggesting that PINK1/Parkin mitophagy is connected with cell death. Similarly, in cardiac microvascular I/R injury [127], PINK1/Parkin mitophagy is elevated as a result of excessive mitochondrial fission. However, inhibition of fission could alleviate PINK1/Parkin mitophagy and thus promote endothelial cells survival [127]. A parallel study verifies the endothelial protective action exerted by Fundc1-induced mitophagy [135]. Post-transcriptional dephosphorylation of Ser13 could activate Fundc1 mitophagy and enhance the resistance of endothelial cells to reperfusion injury [135]. Taken together, PINK1/Parkin and receptor-dependent mitophagy may have different roles in regulating endothelial cell response to I/R injury through undefined mechanisms. Further studies are necessary to figure out the crosstalk between PINK1/Parkin and Fundc1 in regulating mitophagy activity in cardiac microvascular I/R injury.

## 6. Conclusion and Future Perspectives

The entire vascular system, from the heart to the smallest capillary, is lined by endothelial cells. A primary purpose of the microcirculation unit is to couple heart metabolic demand with glucose and oxygen delivery through the blood in a manner dependent on the endothelial-dependent vasodilation [191,192]. Of note, endothelial cells in the heart also adjust vascular resistance appropriately to systemic influences, such as blood gases, pH alterations, circulating hormones, blood pressure fluctuation, and shear stress [193,194]. It is now increasingly appreciated that mitochondria serve as the sentinel organelles that are not only capable of detecting insult signals but also orchestrating inflammation responses [195,196]. Mitochondria play an important role in regulating endothelial cell function (Figure 3). Last but not least, when we think about mitochondrial health and disease, the mitochondrial unfolded protein response (UPR^mt^) has become a hot subject. Although UPR^mt^ is proven to be the downstream event of mitochondrial ROS-mediated oxidative stress including ETC defect and mtDNA damage/mutation, a few studies are available to demonstrate the potential role of UPR^mt^ in endothelial function. Additional investigations are required to describe the molecular mechanism underlying UPR^mt^ in cardiac microvascular I/R injury. The scientific information obtained from mitochondrial dynamics alteration and mitochondrial oxidative stress can be useful for basic and clinically oriented studies, as well as for the development of new diagnostic approaches and tests for cardioprotection strategies.
